# Efficient Biotransformation of Polysialogangliosides for Preparation of GM1 by *Cellulosimicrobium* sp. 21

**DOI:** 10.3390/molecules191016001

**Published:** 2014-10-08

**Authors:** Yan Zheng, Li Ji, Jiayi Leng, Ye Yuan, Honglei Chen, Dongxia Gou, Yufei Gao, Yifa Zhou

**Affiliations:** 1 China-Japan Union Hospital, Jilin University, Changchun 130033, Jilin, China; E-Mail: zhy19780603@aliyun.com; 2 School of Life Sciences, Northeast Normal University, Changchun 130024, Jilin, China; E-Mails: jil132@nenu.edu.cn (L.J.); lengjy366@nenu.edu.cn (J.L.); yuany268@nenu.edu.cn (Y.Y.); chenhl150@nenu.edu.cn (H.C.); goudx479@nenu.edu.cn (D.G.)

**Keywords:** polysialoganglioside, *Cellulosimicrobium* sp. 21, biotransformation, sialidase, GM1

## Abstract

A new ganglioside transformed strain isolated from soil was identified as *Cellulosimicrobium* sp. 21. It produced a sialidase which transformed polysialo-gangliosides GD1 and GT1 into a monosialoterahexosylganglioside,* i.e.*, ganglioside GM1. The sialidase had both NeuAc-α-2,3- and NeuAc-α-2,8-sialidase activity without producing asiolo-GM1. The optimum conditions were evaluated and it was found that the transformation was optimally performed at 30 °C and pH 7.0. The substrate should be added at the beginning of the reaction and the concentration of substrate was 3% (w/v). Under these optimum conditions, *Cellulosimicrobium* sp. 21 converted GD1 and GT1 into GM1 in inorganic medium in a 5 L bioreactor with the recovery rate of 69.3%. The product contained 50.3% GM1 and was purified on silica to give the product with 95% of GM1 with a recovery rate of 30.5%. Therefore, *Cellulosimicrobium* sp. 21 has potential to be applied in the production of GM1 in the pharmaceutical industry.

## 1. Introduction

The gangliosides are a group of complex glycosphingolipids containing sialic acid, existing most prominently in cerebral and nervous tissues. They contain a lipophilic ceramide residue and a carbohydrate chain of diverse length and complexity. The major gangliosides within the gangliotetraose family are classified into GM, GD, GT, and GQ, with one, two, three and four sialic acid groups per molecule, respectively [1].

Gangliosides play an important role in a variety of biological processes involved in the development and maintenance of the brain [2]. GM1 can penetrate the blood-brain barrier and has been developed as a drug applied clinically for neurological disorders such as Alzheimer’s disease, Parkinson’s disease, spinal cord injury, and stroke [3]. GM1 is usually prepared from animal brains by solvent extraction followed by chromatographic purification. Because of its low content in animal brains industrial preparation to isolate GM1 directly is difficult [4,5]. Scientists have made more efforts to convert GD1 and GT1 to GM1 by selectively removing one or two sialic acid residues in order to increase the amount of GM1 [6,7,8]. The methods usually used for this transformation are hydrolysis catalyzed by acids or enzymes. Chemical methods have less selectivity and are highly polluting to the environment, therefore, biotransformations may have more potential in these processes because of their mild conditions, low pollution and high selectivity. Although several sialidases (E.C. 3.2.1.18) which transform GD1 and GT1 to GM1 are available commercially, such as sialidase from *Clostridium perfringens* (*C. welchii*) Type VI and α-(2-3,6,8,9) sialidase from *Arthrobacter ureafaciens*, the high cost of their production limits their application in industry. Thus, the use of sialidase-producing microorganisms to convert GD1 and GT1 into GM1 has caught researchers’ attention. Some strains such as *Pseudomonas* sp. strain YF-2 [9], *Brevibacterium casei* [10,11] and *Oerskovia xanthineolytica* YZ-2 [12] have been found to produce sialidases which convert GD1 and GT1 to GM1, but the transformation efficiency and conditions still hinder their application in industry. It is necessary to find more microbes for the efficient transformation of GD1 and GT1. In this study, we reported a new strain of *Cellulosimicrobium* isolated from soil, which could covert polysialogangliosides to GM1 with more efficiency. Further, the optimum conditions for the transformation were evaluated and the products were identified.

## 2. Results and Discussion

### 2.1. Screening of Microorganisms for Biotransformation

By screening microbes isolated from soil with monitoring by TLC and HPLC analysis, we found four stains that could convert polysialogangliosides into GM1 (Table 1). As shown, the bacterium numbered 21 (No. 21) showed higher catalytic activity in the transformation of GD1 and GT1 into GM1 than others. But there was little digestion of GM1 after incubation for three days, indicating that No. 21 could not hydrolyze GM1 to asialo-GM1. Strain No. 21 was identified by 16S rDNA gene sequence analysis by Shangon Biotech Co. Ltd. (Shanghai, China), as *Cellulosimicrobium*, and named *Cellulosimicrobium* sp. 21. The 16S rDNA (1407 bp) sequence analysis was uploaded in GenBank (accession number: KF133829).

**Table 1:** Screening results of microbiotransformation of polysialogangliosides.

Strain	Content of GM1 (w/w, %)	Conversion Time (h)
No. 2	45.5	72
No. 4	37.0	72
No. 5	46.1	48
No.21	46.2	20

**Figure 1:**
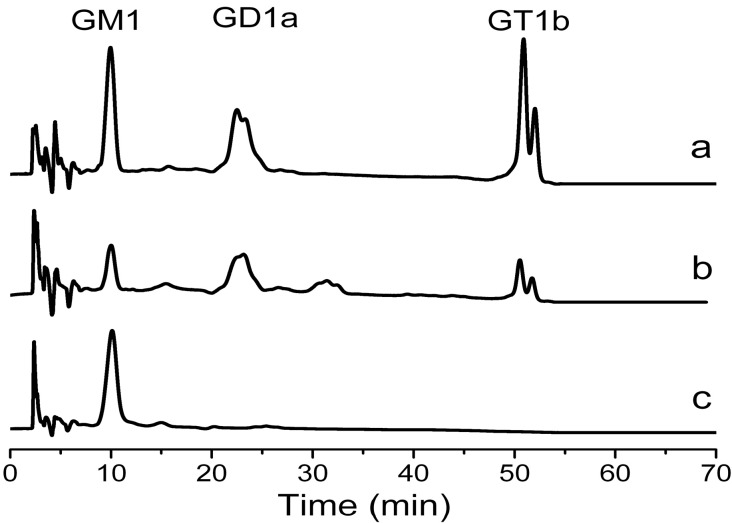
HPLC analysis of biotransformation product. Standards (**a**); gangliosides mixture (**b**); biotransformation product (**c**). Eluent: acetonitrile-phosphate buffer. Flow rate 1 mL/min at 20 °C and detection 215 nm.

Three kinds of microorganisms have been reported for GM1 biotransformation [9,10,11,12]. Two of them used a transformed organic medium [10,12], and one used an inorganic medium. *Pseudomonas* sp. strain YF-2 was the first microorganism which was reported for GM1 biotransformation by Kyushu University [9]. The substrate which was used contained GD1a, GD1b and GT1b. It took 3 days to finish the transformation in inorganic medium at 25 °C. The rate of biotransformation was 80%–90%. The other two were both reported by East China University of Science and Technology. Both of the substrates were crude gangliosides which contained GM1, GD1a, GD1b and GT1b. *Brevibacterium casei* finished the transformation in the medium with yeast powder at 30 °C after 24 h [10,11]. *Oerskovia xanthineolytica* YZ-2 finished the transformation in the medium with yeast powder at 37 °C after 18 h. The content of GM1 was increased from 9% in crude gangliosides to 45% with a yield of 70% (w/w) [12]. Comparing to those microbes reported in the literature, the No. 21 strain was more efficient. No. 21 almost completely transformed GD1 and GT1 to GM1 in inorganic medium after 20 h. The content of GM1 was increased from 11.1% to 46.1% with a yield of 72.2%. Nearly all the GD1 and GT1b were transformed to GM1 at the end of biotransformation (Figure 1). Next the optimal conditions for the biotransformation were studied to increase the transformation rate.

### 2.2. Optimization of Conditions for Biotransformation

In order to apply this biotransformation to the preparation of GM1, we evaluated the effects of the time of substrate addition, extra carbon supplements, temperature, pH and substrate concentration on biomass of microorganisms and the sialidase activity.

#### 2.2.1. Effect of Carbon Sources on Biotransformation

**Figure 2:**
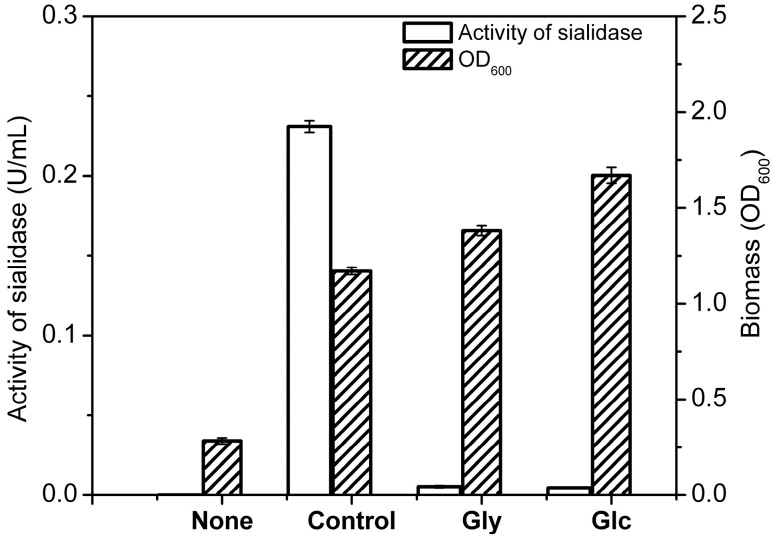
Effect of carbon source on transformation activity of *Cellulosimicrobium* sp. 21. Activity of sialidase (blank column), biomass (filled column). None, without carbon source; GLS, 1% polysialogangliosides; Gly, 1% polysialogangliosides and 1% glycerol; Glc, 1% polysialogangliosides and 1% glucose. Biomass was determined by the absorption at 600 nm. Activity of sialidase was determined by using 4-MU-NeuAc as fluorescent substrate and detection at 335 nm/460 nm.

In order to evaluate the effects of carbon sources on the growth of *Cellulosimicrobium* sp. 21 and its transformation activity, we cultured *Cellulosimicrobium* sp. 21 in medium containing polysialo-gangliosides as sole carbon source or the mixture of polysialogangliosides with glucose or glycerol (1%, w/v) as carbon source. As shown in Figure 2, as expected *Cellulosimicrobium* sp. 21 in control (None) grew only a little and produced no sialidase activity; it grew well and produced very high salidase transformation activity when polysialogangliosides were used as the sole carbon source (GLS); although glucose or glycerol increased the biomass, they decreased the sialidase activity (Gly and Glc). These results indicated that the extra carbon sources hindered salidase production. The sialidase was produced better from polysialogangliosides without other carbon sources, therefore, polysialo-gangliosides could be effectively transformed by *Cellulosimicrobium* sp. 21 when they were sole carbon source.

#### 2.2.2. The Optimal Time for Adding Substrate and Concentration of Substrate

**Figure 3:**
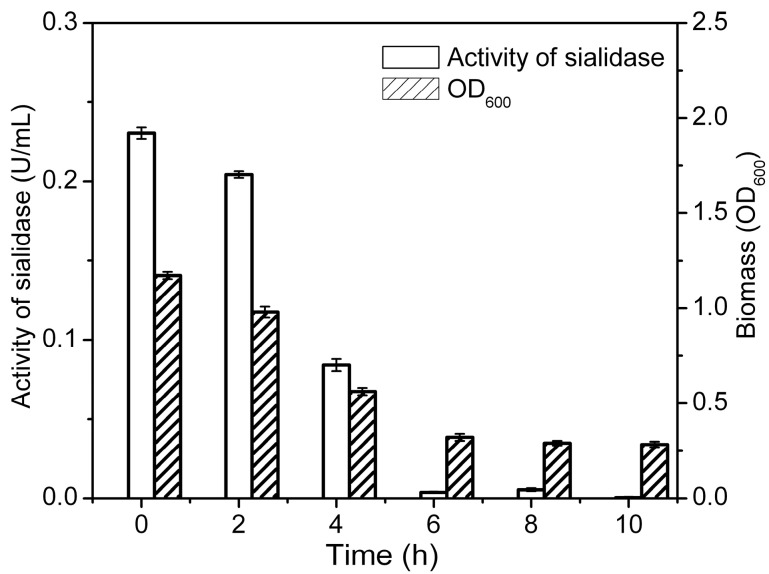
Effect of substrate addition time on biotransformation. Activity of sialidase (blank column), biomass (filled column). Biomass was determined by the absorption at 600 nm. Activity of sialidase was determined by using 4-MU-NeuAc as fluorescent substrate and detection at 335 nm/460 nm.

**Figure 4:**
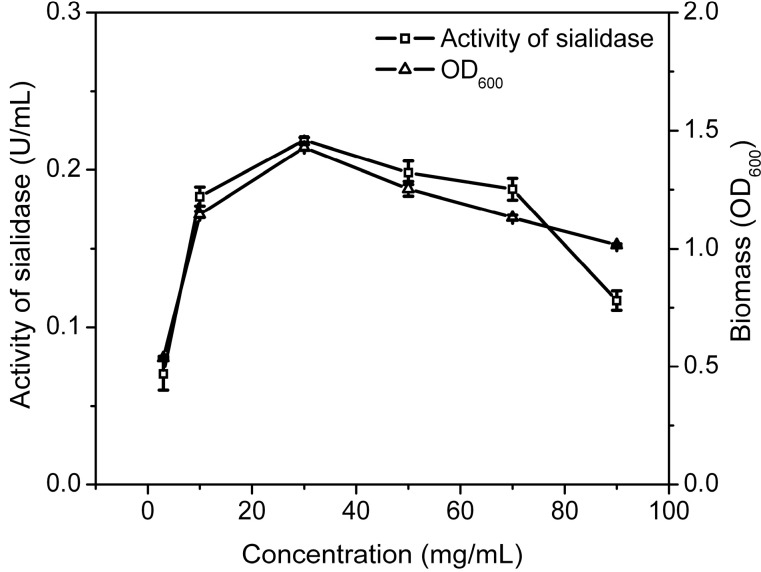
Effect of substrate concentration on biotransformation of *Cellulosimicrobium* sp. 21. Activity of sialidase (squares), Biomass (triangles). Biomass was determined by the absorption at 600 nm. Activity of sialidase was determined by using 4-MU-NeuAc as fluorescent substrate and detection at 335 nm/460 nm.

To test the optimal time for adding gangliosides, *Cellulosimicrobium* sp. 21 was cultured in medium with gangliosides as sole carbon source. The gangliosides were added to the medium at 0, 2, 4, 6, 8, 10 h. As shown in Figure 3, the activity of sialidase and the biomass of *Cellulosimicrobium* sp. 21 decreased significantly when ganglioside was added after 2 h, indicating that the optimal time of adding the gangliosides mixture was at the beginning of the biotransformation. To evaluate the effect of the concentration of substrate, 0.5%, 1%, 3%, 5%, 7% and 9% of ganglioside in the medium were used to cultivate *Cellulosimicrobium* sp. 21 at 30 °C for 20 h. As shown in Figure 4, the highest sialidase activity is observed in 3% of substrate.

#### 2.2.3. The Optimal Biotransformation Temperature and pH 

**Figure 5:**
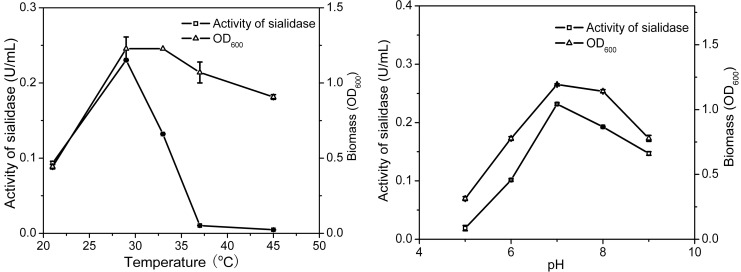
Effect of temperature and pH on biotransformation activity of *Cellulosimicrobium* sp. 21. Activity of sialidase (squares), Biomass (triangles). Biomass was determined by the absorption at 600 nm. Activity of sialidase was determined by using 4-MU-NeuAc as fluorescent substrate and detection at 335 nm/460 nm.

To evaluate the effects of temperature on the transformation, *Cellulosimicrobium* sp. 21 was cultured at different temperatures and then the biomass and the activity of sialidase were tested. As shown in Figure 5, both the biomass and the activity of sialidase achieved the highest values at 30 °C, indicating that the optimal temperature for transformation was 30 °C. *Cellulosimicrobium* sp. 21 was incubated in different medium pH values (from 5 to 9) and then the growth and sialidase activity were determined. Figure 5 shows the effect of pH on the cell growth and the activity of sialidase. Both the biomass and the activity of sialidase achieved their highest values at pH 7, indicating that pH 7 was the optimal condition for the transformation.

### 2.3. Scale-Up of Conversion from Polysialogangliosides to GM1 in 5 L Bioreactor

According to the optimal conditions evaluated in flask, the biotransformation of GD1 and GT1 to GM1 was performed in a 5 L bioreactor. After 20 h transformation, the product was centrifuged at 3200 *g* for 10 min, dialyzed against distilled water and freeze dried, with a yield of 69.3%. The content of GM1 in the product reached 50.3%.

**Figure 6:**
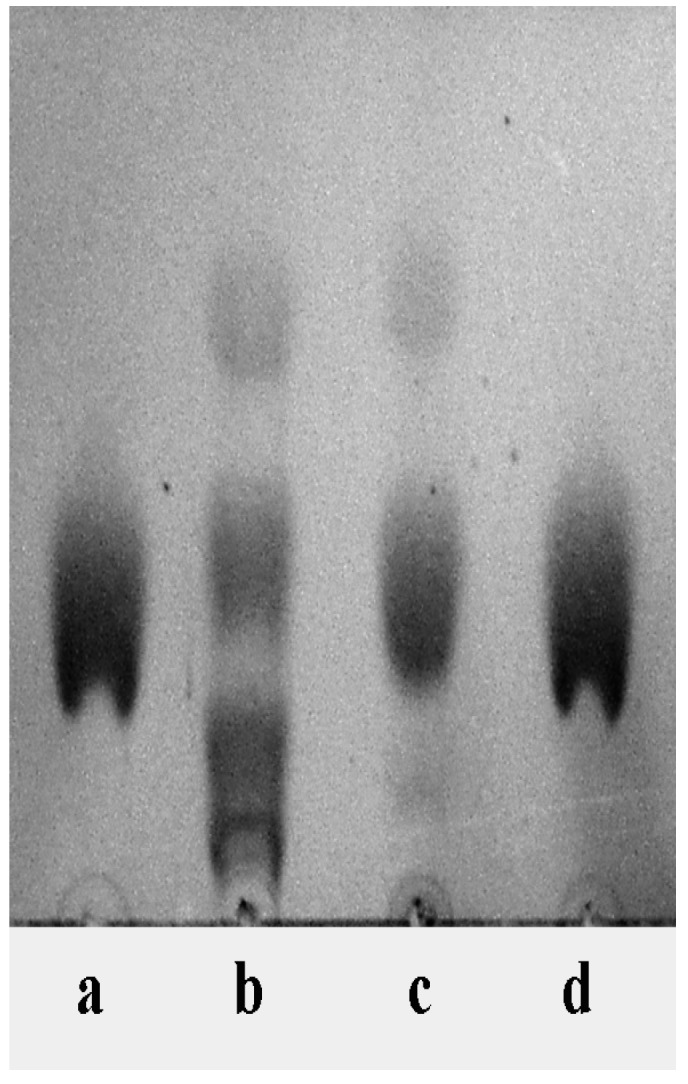
Result of the purification detected by TLC. Standard GM1 (**a**); ganglioside mixture (**b**); product after biotransformation (**c**); sample after purification (**d**). Solvent system: chloroform-methanol-0.02% calcium chloride aqueous solution (60:36:8, v/v/v).

**Figure 7:**
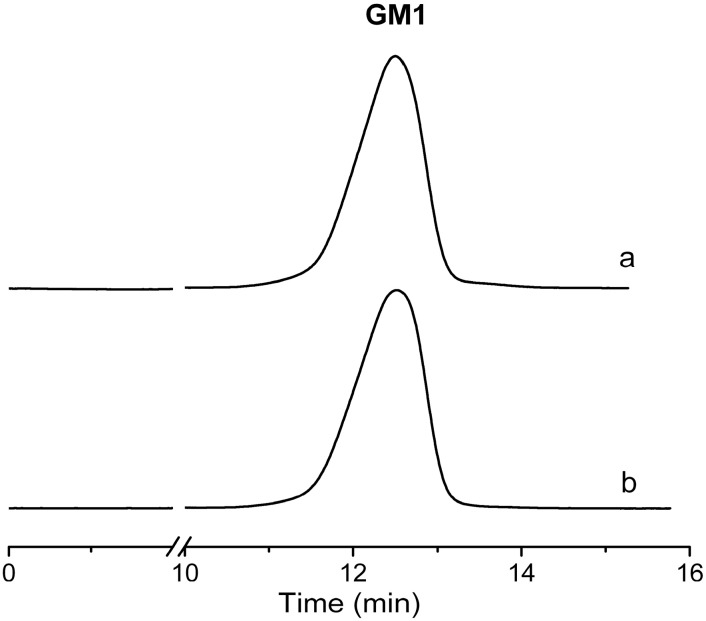
Result of GM1 purification. Standard of GM1 (**a**); sample after purification (**b**). Eluent: acetonitrile-phosphate buffer. Flow rate 1 mL/min at 20 °C and detection 215 nm.

The biotransformation occurred in a simple inorganic medium with high efficiency; the purification of GM1 was relatively simple. GM1 was purified by silica chromatography only with a recovery rate of 30.5% and the purity was more than 95% (Figure 6 and Figure 7). The purification of GM1 from biotransformation systems in different ways has been reported. For *Pseudomonas* sp. strainYF-2, GM1 was purified from the culture supernatant by C18 reverse-phased chromatography, followed by DEAE-Sephadex A25 anion-exchange chromatography [9]. For *Brevibacterium casei* and *Oerskovia xanthineolytica* YZ-2, GM1 was purified from the culture supernatant by partitioning with a chloroform and methanol mixture, followed by acetone precipitation and silica gel chromatography [10,12]. The purification procedure was necessary to get rid of the residues of other gangliosides in the system, especially GD1a, GD1b and GT1b and the organic medium used in the biotransformation system. The biotransformation reported in this paper was very efficient and the products did not contain GD1a, GD1b and GT1b. In addition, the medium had a quite simple composition. Therefore, the purification of GM1 was relatively simple, requiring only silica gel chromatography.

### 2.4. Analysis of GM1

**Figure 8:**
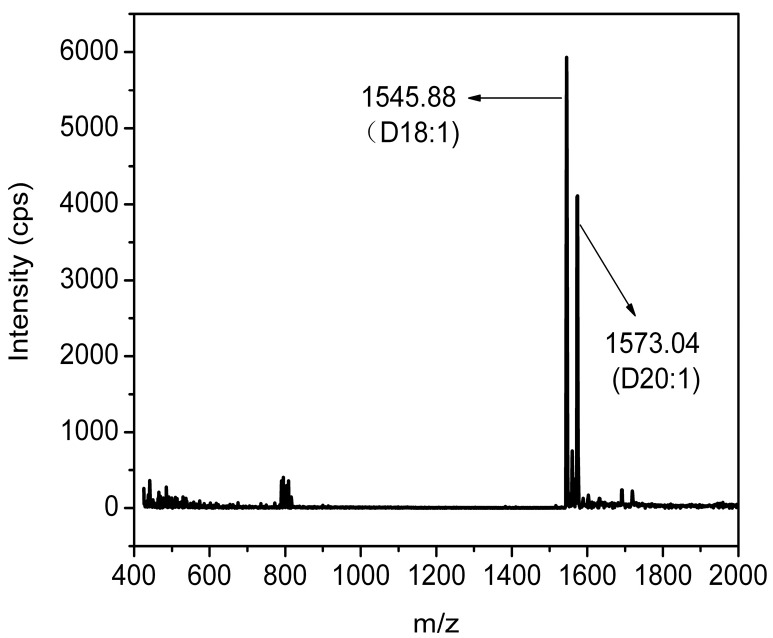
The result of ESI-MS. The sample was dissolved in MeOH and detected in the negative-ion mode.

**Figure 9:**
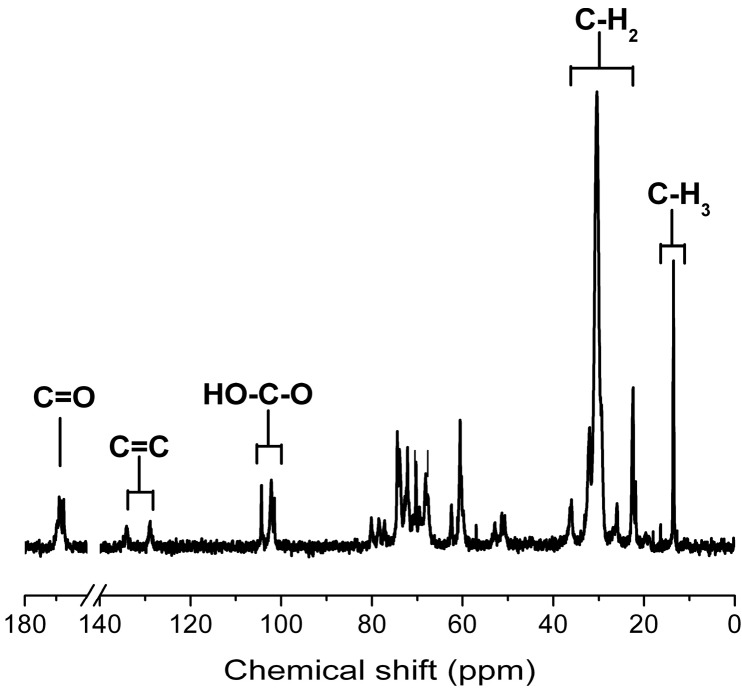
The result of ^13^C-NMR. The sample was dissolved in D_2_O and detected at 150 MHz.

The final product GM1 was identified by ESI-MS and ^13^C-NMR. The characteristic negative ions (M−H)^−^ were found at *m/z* 1545.88 and 1573.04, which correspond to two molecular species of the monosialoganglioside GM1 with different ceramide (Figure 8). The ^13^C-NMR was recorded with purified GM1. The major chemical shifts are shown in Figure 9 and listed in Table 2. Compared with the results which have been reported in the literature, the final product was confirmed as GM1.

**Table 2:** ^13^C-NMR data for GM1 (150 MHz, solvent: D_2_O).

Residue	Carbon	Chemical Shift (ppm)
β-d-Glc	C-1	102.14
	C-4	78.49
β-d-Gal	C-1	102.17
	C-3	73.91
	C-4	77.19
α-d-NeuAc	C-1	174.51
	C-2	101.49
	N–C=O	174.23
β-d-GalNAc	C-1	101.99
	C-3	80.09
	N–C=O	173.78
β-d-Gal	C-1	104.36
Ceramide	C-1-3	51.31–68.16
	C-4	134.06
	C-5	128.89
	C-6-17	21.83–30.40
	C-18	13.60
Fatty acid chains	N–C=O	173.78
	C-2-15	21.83–30.40
	C-16	13.60

## 3. Experimental Section

### 3.1. Materials

Ganglioside mixture containing GT1, GD1 and GM1 was extracted from pig brain as described in the literature [5]. Standards of GM1, GD1a, GT1b and 2'-(4-Methylumbelliferyl)-α-d-*N*-acetyl-neuraminic acid sodium salt hydrate (4-MU-NeuAc) were purchased from Sigma-Aldrich (St. Louis, MO, USA). All other reagents and chemicals were commercially available reagents of analytical grade produced in China.

### 3.2. Analytical Methods

Thin layer chromatography (TLC) was carried out using a silica gel G 60 plate with the solvent system: chloroform-methanol-0.02% calcium chloride aqueous solution (60:36:8, v/v/v). The gangliosides developed on the plate were stained by spraying with resorcinol-HCl reagent, and then heating at 115 °C for 15 min [13]. The content of GM1 was determined by HPLC (Shimadzu, Kyoto, Japan) with an analytical Inertsil NH_2_ column (4.6 × 250 mm, 5 μm, Shimadzu, Kyoto, Japan) as described by Gandini* et al.* [14]. ^13^C-NMR spectra were recorded on a Bruker AV 600 NMR spectrometer (Bruker, Benutzertagung Ettlingen, Germany) at 150 MHz with D_2_O as the solvent. The electrospray ionization (ESI)-MS analysis were performed by the API 2000 LC/MS/MS system (Applied Biosystems, Foster City, CA, USA) as described before [15,16].

### 3.3. Strain Screening

Soil samples collected from Changbai Mountain (Fusong, Jilin, China) were diluted with de-ionized water and centrifuged to remove particles. Then, the suspension were moved to a 1000 mL flask with 200 mL enrichment medium (NaH_2_PO_4_·2H_2_O, 3.0 g/L, Na_2_HPO_4_·12H_2_O, 10.9 g/L; (NH_4_)_2_SO_4_, 10.0 g/L; pH 7.0) with 1% gangliosides mixture (w/v), and incubated at 30 °C, 200 rpm for 24 h to enrich microorganisms. The broth of the microorganisms was checked by TLC at intervals.

The microorganisms screened above were streaked on LB agar plates. The isolated strains from LB agar plates were reexamined by incubating in enrichment medium with gangliosides mixture (1% w/v). GM1 contents after biotransformation was taken by HPLC. The strain having the highest activity of transformation was identified further by 16S rDNA gene sequencing.

### 3.4. 16S rDNA Gene Sequencing

Chromosomal DNA of isolated strain for PCR was extracted by the method of Pospiech and Neumann [17]. 16S rDNA was cloned from chromosomal DNA using oligonucleotide primers: primer 1, 5'-AGAGTTTGATCCTGGCTCAG-3'; primer 2, 5'-GGTTACCTTGTTACGACTT-3'. The program for PCR was following: 94 °C 5 min, 29 cycles of three steps (94 °C for 30 s, 54 °C for 30 s and 72 °C for 90 s), and followed by 72 °C for 10 min additionally.The amplified DNA sequence was analyzed by Sangon Biotech Co. Ltd. (Shanghai, China).

### 3.5. Enzymatic Activity Assay of Sialidase

Sialidase activity was determined by using 4-MU-NeuAc as fluorescent substrate. The reaction system was 200 μL with 10 μL 1 mmol/L 4-MU-NeuAc, 10 μL sample solution, and 80 μL of 200 mmol/L pH 5.5 sodium acetate buffer. The reaction system was used to measure fluorescence (335 nm/460 nm) after incubation at 37 °C for 10 min and quenching with 1 mol/L-glycine-sodium hydroxide buffer solution (200 μL, pH 10.4) addition. The control of the reaction system was boiled sample. One unit of the activity was defined as the amount of sialidase that hydrolyzed 1 μmol substrate per minute [18].

### 3.6. Cultivation and Biotransformation

Cells were inoculated in a 25 mL flask with 10 mL LB medium at 30 °C, 200 rpm for 16 h, and then transferred (5% inoculation) into 10 mL enrichment medium containing 1% gangliosides mixture in a 25 mL flask for sialidase production at 30 °C, 200 rpm for 20 h. Both samples were collected for GM1 content and activity of sialidase determination during the biotransformation. The optimum conditions of biotransformation were tested, including the carbon source, the optimal time for adding substrate and concentration of substrate, the optimal temperature and pH.

### 3.7. Preparative Scale Biotransformation

The biotransformation for preparation of GM1 was enlarged in a bioreactor (BIOTECH-5BG, Shanghai, China) equipped with DO and pH electrodes. Enrichment medium (2 L) was used for cell culture and GM1 conversion after sterilization at 115 °C for 20 min. The *Cellulosimicrobium* sp. 21 was inoculated in 500 mL flask with 100 mL LB medium at 30 °C, 200 rpm for cultivating 16 h, and then transferred (5% inoculation) in bioreactor with 2 L enrichment medium containing 3% gangliosides mixture (containing 11% of GM1) extracted from pig brains. The medium was inorganic medium only with gangliosides. The conditions of transformation were at 30 °C, pH 7.0, 200 rpm and 0.8 L/min of air supplement. After 20 h transformation, the product was centrifuged at 3200× *g* for 10 min, dialyzed against distill water and freeze dried.

### 3.8. Separation and Purification of GM1

When the biotransformation was finished, cells were removed by centrifugation at 3200×* g* for 10 min. The supernatant was dialyzed against distilled water and freeze dried. The content of GM1 in the product was determined by HPLC. The purification of GM1 was performed by the method of Ledeen and Yu [19] with some modifications. The biotransformation product was dissolved in chloroform-methanol (4/1, v/v) and subjected to silica gel chromatography by first washing the column with chloroform-methanol (4/1, v/v) to remove impurities, then eluting with chloroform-methanol (5/4, v/v) to give GM1.

## 4. Conclusions 

A novel strain for GM1 production was isolated and named *Cellulosimicrobium* sp. 21. It can transfer polysialoganglioside to GM1 in simple inorganic medium and under mild conditions. The yield (69.3%) was higher than those of biotransformations reported in the literature [9,10,11,12]. The biotransformation in simple inorganic medium resulted in a simpler product purification than those of similar biotransformation products in usual media. The purification was performed by chromatography on silica gel to get GM1 of over 95% purity. Therefore, the method developed in this paper has potential for preparation of GM1 in industry.
